# An exploration into the efficacy of public warning signs: A zoo case study

**DOI:** 10.1371/journal.pone.0207246

**Published:** 2018-11-09

**Authors:** Ellie N. Parker, Lauren Bramley, Laura Scott, Andrew R. Marshall, Katie E. Slocombe

**Affiliations:** 1 Department of Psychology, University of York, York, United Kingdom; 2 Craven College, Skipton, United Kingdom; 3 Flamingo Land Ltd., Kirby Misperton, United Kingdom; 4 CIRCLE, Environment Dept., University of York, York, United Kingdom; 5 Tropical Forest and People Research Centre, University of the Sunshine Coast, Sippy Downs, Queensland, Australia; Middlesex University, UNITED KINGDOM

## Abstract

Unauthorised feeding and touching of the animals by visitors to zoos and wildlife parks pose serious threats to the health of both animals and visitors alike. We tested the efficacy of four different “do not feed” signs designed to prevent zoo visitors from feeding a group of meerkats. Signs consisted of one of two different written messages and imagery of either a pair of watching human eyes, or meerkat pawprints as a control. Covert observation of visitor behaviour in the presence and absence of the signs was analysed. Visitors were significantly less likely to feed the meerkats when signs were present, than when they were absent. The effect of the signs was specific to the targeted behaviour in that feeding was reduced, but attempts to touch the meerkats increased with the presence of the signs. We did not find that the presence of watching eyes or the different wording on the signs affected the likelihood of visitors feeding the meerkats. We also examined factors that influenced the likelihood of visitors attending to the signs. We found that children were more likely to attend to signs than adults which has important implications for the design of such signs. Together our findings show that signs are effective in reducing the unwanted behaviours they target but may also result in displacement of these negative behaviours and that children are more likely to attend to these signs than adults.

## Introduction

Zoos provide a unique opportunity for visitors to interact with wild and exotic animals. Educational demonstrations, animal rides, public feeding and children’s zoos have long since been commonplace in zoos to attract and engage visitors [1]. Direct contact is a particular draw for visitors, with research finding 65% of teachers in a survey considered touching the animals important when visiting with students [2]. However, zoonotic disease transmission poses risks for humans who make contact with animals [3]. Additionally, visitors may detrimentally affect the animals themselves as proximity to, and interactions with, humans can induce stress [4].Audience induced stress might, however, be mitigated by chronic exposure in some primate species, and visitors throwing food to primates could have been enriching [5]. However, unauthorised feeding poses serious nutritional risks for the animals’ health such as increased chance of obesity and diabetes [5]. It is important to explore how visitor behaviour can be changed so that the risks to both visitors and animals that are associated with unauthorised feeding and physical contact can be effectively reduced.

“Do not feed” signs are one of the oldest forms of signs found in zoos [6] and play a vital role in promoting zoo inhabitants’ welfare. An investigation into the efficacy of “do not feed” signs found no difference in levels of feeding between no sign and a simple, instructional sign (“Please do not feed the animals”). However, an explanatory sign (“Please do not feed. These animals are on special diets”) significantly reduced feeding in a monkey exhibit [7]. This highlights the need to understand the aspects of sign design that influence the efficacy of the signs. In terms of wording, research suggests that less may be more as signs with fewer words are read by more people while longer signs attract less attention [6]. However, once a message has been attended to, even if it is not read carefully, increasing the number of arguments in a message can still increase its persuasiveness [8].

Simply changing words in economic decision problems can influence peoples’ decisions by suggesting how they should respond [9]. Compliance with sign messages can also be greatly influenced by the wording and framing of the message. In environmental research, research has found a negatively worded message (“please do not remove petrified wood from the park”) rather than a positively worded message (“please leave petrified wood in the park”) was more successful at deterring people from stealing wood [10]. A variety of other studies also support a negativity bias, where negative framing increases cooperation with the message. Health psychology research suggests that participants are more likely to perform testicular and breast self-examinations when a message is negatively framed [11, 12]. Negatively framed messages seem to be most effective at influencing behaviour when individuals are already interested in the issue: one study found a negatively framed message encouraging animal adoption was significantly more effective than a positively framed message, but only with individuals who were already interested in animal adoption [13].

The degree to which a sign engages the audience can also be affected by its perceived relevance. Previous research outlines the importance of framing the message personally toward the current reader to maximise its relevance [14]. Simply using personal pronouns can also make a message more direct and increase its significance to the audience [6]. Attitudes and behaviours are most likely to change if readers are illustrated as the audience who will be personally affected by their actions [15].

It is clear that the wording of sign messages affects the likelihood of audiences attending to and complying with them, but imagery has also been shown to affect a sign’s efficacy. Pictures closely related to the accompanying text can significantly increase attention and recall of the related message [16] and are often more universally understood than text [17]. Pictograms can therefore be especially useful for zoo visitors with lower literary abilities such as children [18].

The application of ‘watching’ eyes to messages has been shown to increase prosocial behaviours and adherence to messages prohibiting negative behaviours in a variety of contexts by provoking reputational concerns (e.g. [19, 20]). For instance, signs with eyes displaced over 60% of bike theft on a university campus from experimental locations to control locations nearby [21]. Another line of research into prosocial behaviour found prosociality in an anonymous, one-shot game to be driven by a preference to do the right thing morally and that this preference to ‘do good’ was as strong as the preference to avoid doing wrong [22, 23].

The current study aimed to test the efficacy of “do not feed” signs at deterring zoo visitors from engaging in unauthorised feeding or touching of animals and additionally whether different sign wording and the presence of watching eyes affected their efficacy or the attention they drew from zoo visitors. Covert observations of visitor behaviour surrounding a meerkat enclosure were conducted in the absence or presence of signs with varying wording and imagery. We used four signs with a unique combination of wording and imagery. We predicted, in line with previous findings [7], that there would be a significant reduction in feeding when a sign was present compared to absent. Given the specificity of the sign messages to feeding, it was predicted that there would be no significant difference in the levels of trying to touch the meerkats when the sign was present and absent.

In terms of sign design, it was predicted that there would be a significantly lower proportion of visitors feeding meerkats when signs with a longer explanatory text [7] and personal wording [14] were present compared to the signs with shorter, non-personal text. In line with previous findings [21, 24, 25], we predicted there would be a significantly lower proportion of visitors feeding and trying to touch meerkats when signs with watching eyes versus a control image were present. Although the sign doesn’t address touching the meerkats, previous research has found a sign with watching eyes reduced littering even with an unrelated message [25], thus the watching eyes may also reduce trying to touch the animals which we assumed visitors knew they should not be engaging in.

We examined various factors that might have affected whether visitors overtly paid attention to the sign. As several studies have suggested that sign readers are usually adults rather than children [6, 26], we predicted that more adults than children would attend to the signs. In line with previous research [6] we predicted that more attention would be paid to signs containing the shorter message and that watching eyes may capture attention more than pawprints due to the salient nature of eyes as important social stimuli.

## Methods

### Ethics statement

Both the University of York Department of Psychology and Department of Biology Ethics Committees approved the study, including covert observation and the concept for the study was also approved by the Flamingo Land Ethics Committee. Informed consent could not be obtained without affecting people’s behaviour, however signs were displayed at the park entrances highlighting that observations of visitors were being conducted and all behaviour was observed in a public area where visitors could be expected to be observed by others. The individual in the photos used for the watching eyes in this manuscript has given written informed consent (as outlined in PLOS consent form) to publish this photograph. The data for the current study is available to download from the York Research Database (https://doi.org/10.15124/cf5a42f1-8e2e-4b10-bc75-deea086a01ee).

### Study site

Flamingo Land Theme Park and Zoo (hereafter “Flamingo Land”) was the 4th most visited paid attraction in England in 2016 having experienced 1,610,556 visitors [27]. This study focussed on a meerkat enclosure where animal keepers anecdotally reported high levels of unauthorised feeding. There was no existing signage on the enclosure studied.

### Participants

#### Visitors

Participants were observed covertly by experimenters dressed in inconspicuous clothing. Signs at Flamingo Land’s entrance informed visitors that observations may be taking place and all observations occurred in a public place where one might expect to be observed by other visitors and zoo personnel.

#### Meerkats

Three meerkats aged 8–9 years old lived in the study enclosure (2 males, 1 female). The meerkats were fed a specialised diet twice daily by keepers. During observations they could access an inside area and an outside enclosure where an approximately 1m high wall separated the meerkats from visitors. Observations of all three outdoor enclosure walls available to visitors could be made from one position enabling experimenters to take all occurrence recordings of visitor behaviour.

### Materials

**Fig 1:**
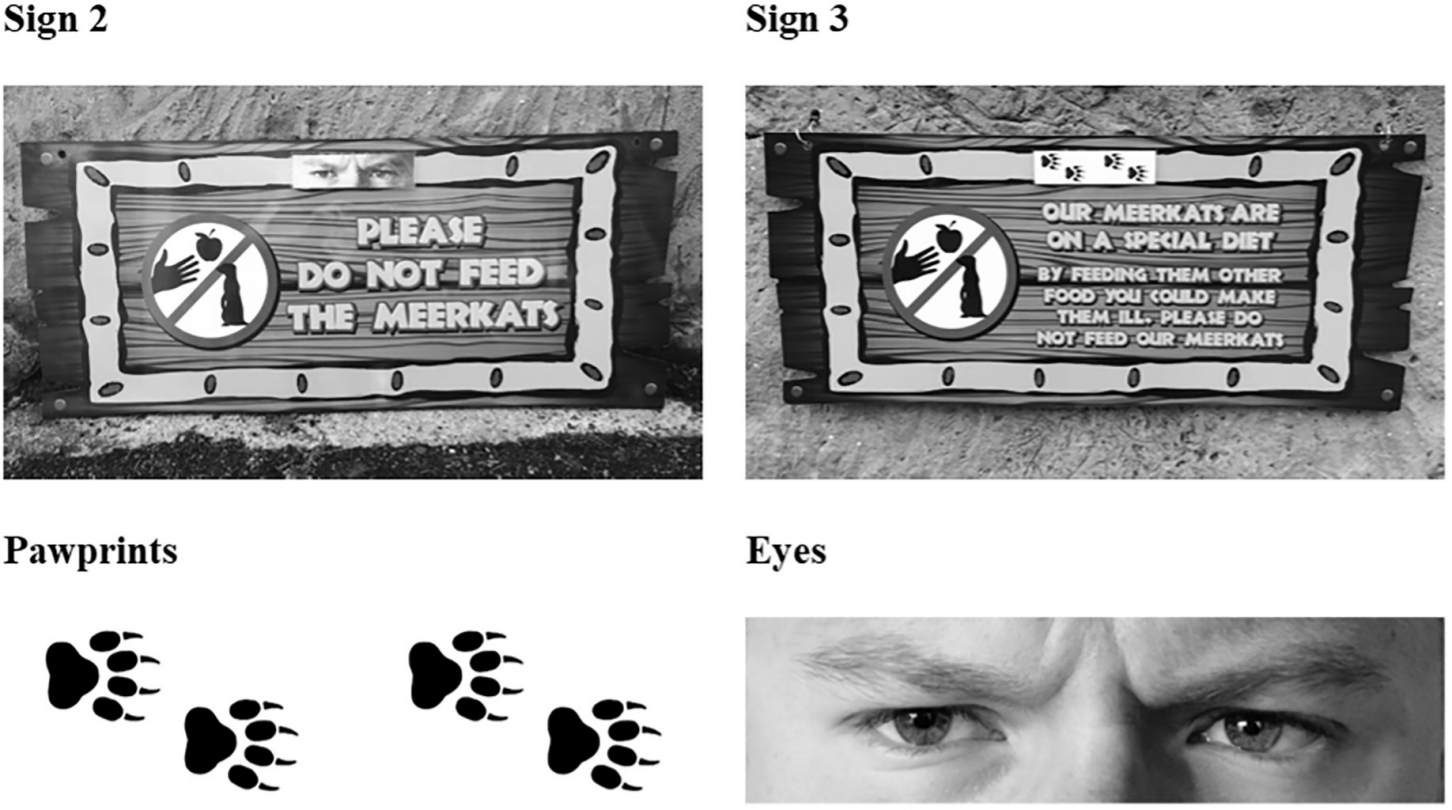
Photographs of signs 2 and 3, illustrating the two types of wording used and images of eyes and pawprints used respectively as the treatment and control. The signs had a brown wood-effect background with yellow borders and text. The eyes and pawprints were greyscale.

As observations were covert, data were noted in spreadsheets on the Google Sheets app using the experimenter’s iPhone SE. One sign was placed in the centre of each of the three enclosure walls the visitors had access to, and were produced in the style of all Flamingo Land’s signs (Fig 1). The signs all contained a pictogram with a hand, apple and meerkat silhouette within a prohibited action image (Fig 1), based on previous research suggesting the most effective “do not feed” sign pictograms contain a hand, food item and animal [28]. Each of the four signs also contained a fully factorial combination of two forms of wording and imagery (Table 1; Fig 1). The watching eyes and pawprints images (Fig 1) were 15cm wide and 4cm tall and were laminated and attached with Velcro.

**Table 1:** The wording and imagery used for each sign.

Sign	Wording	Imagery
1	Please do not feed the meerkats	Pawprints
2	Please do not feed the meerkats	Eyes
3	Our meerkats are on a special diet By feeding them other food you could make them ill, please do not feed our meerkats	Pawprints
4	Our meerkats are on a special diet By feeding them other food you could make them ill, please do not feed our meerkats	Eyes

### Design

A between-subjects design was used assuming visitors recorded within and between each of the different conditions were independent, thus each visitor only experienced one sign or baseline condition.

### Procedure

All data were collected on weekdays between 11:30am and 2:30pm during the school summer holidays as this was the busiest time of day and year, thus maximising the sample size. Baseline data (no sign) were collected for 10 days and following this, the four sign designs were rotated for 10 days, whereby each day a different sign design was used. Signs were installed prior to the start of each observation session. On eight days the same sign remained for the whole observation period, but on two days the signs were changed part way through to equalise the amount of observation with each type of sign.

### Data collection

Data were collected by two experimenters, only when the meerkats were in their outdoor enclosure, and hence only where direct interaction between meerkats and visitors was possible. We sampled 9813 visitors over the course of 60 hours between the dates of 25th July and 23rd August 2016. We collected data in 30 minute sample periods, during which we recorded the number of visitors who visited the meerkat enclosure. We only included visitors taller than the height of the wall (e.g. excluding small children) who stood within 1m of the meerkat enclosure for 10 seconds or more as having ‘visited’ the enclosure. We recorded only visitors who were above 1m tall to ensure that they exceeded the height of the perimeter wall (and hence had full view of the meerkats), and to place focus on individuals who were potentially old enough to understand the signs. Within the 30 minute sample period we also recorded all occurrences of visitors feeding or trying to touch the meerkats. When a feeding event or touching attempt occurred, we recorded the time of the event and the age category (adult/child/both) and perceived gender (male/female/both) of the visitors involved in the event (definitions in Table 2).

**Table 2:** Definitions of data recorded for each visitor.

Data	Definition
Feeding	Throwing any food item or object into the enclosure or offering it to a meerkat, i.e. lowering the object over the enclosure wall and presenting it towards the meerkat(s).
Trying to touch	Anyone attempting to touch or touching the meerkats by leaning over the wall and reaching towards the meerkats.
Age category	”Children” were people judged to be less than 16 years old. If a child was held up by an adult and subsequently fed or tried to touch the meerkats, the pair were classed as “both”.
Perceived Gender	Visitors were judged by experimenters on the basis of their appearance to be either male or female. If an adult held up a child and the child was perceived to be a different gender to the adult, the pair were classed as “both”.

On days where the signs were present, we also conducted focal sign observations. The focal sign area was the area within which we deemed that the sign could be easily read. This comprised a 4.66m diameter rectangular area around the sign. Visitors were included in the focal sign sample if they stood within 1m of the wall and within the focal sign area, for 10 seconds or more. We recorded their age category (adult/child) and perceived gender (male/female) (Table 2) and whether they ignored or acknowledged (pointed at, read aloud or commented on the sign) the sign.

### Statistical analysis

Generalized Linear Mixed effects Models (GLMMs) with a binomial error structure were used to investigate the influence of categorical explanatory variables (e.g. sign imagery, sign wording) on a binary response variable (e.g. feed meerkats or not). These models were implemented in R (version 3.5.0) [29] using package lme4 [30]. As we sampled the behaviour of multiple people within each day and 30-minute sample period, we fitted ‘day’ and ‘sample period’ as random factors [31] by conducting random intercepts models using the package lme4 [30]. To assess the significance of a full model we compared it to a null model comprising only the intercept and random effects, using a likelihood ratio test [32]. For models containing multiple explanatory factors, we assessed the significance of each factor by comparing the full model containing the factor to a reduced model without that factor, using a likelihood ratio test.

To assess the effect of sign presence on visitor behaviour models were run where each individual who visited the meerkat enclosure was entered as a data point (N = 9813). To assess the effect of sign design on visitor behaviour models were run where each individual who visited the meerkat enclosure when a sign was present was entered as a data point (N = 4655). Finally to assess the factors that may influence whether or not a visitor attended to the sign models were run where each individual who entered the focal sign area was entered as a data point (N = 2254).

## Results

A continuous count of visitor arrivals showed 9813 people stopped at the meerkat enclosure studied during our observations. Over the course of 20 observation days, 156 occurrences of feeding and 331 instances of trying to touch were recorded. Table 3 shows that roughly equal percentages of males and females and adults and children were involved in these events, so these events are not being predominantly perpetrated by a single class of visitor. As it was not possible to record the age class and perceived gender of all visitors in the continuous visitor count, we cannot assess if the observed distribution of negative behaviour was different from the expected distribution.

**Table 3:** The percentage of feeding and trying to touch meerkats events perpetrated by each type of visitor.

		Feeding (N = 156)	Trying to touch (N = 337)
**Age**	Child	42.31%	48.37%
	Adult	51.92%	51.63%
	Both	5.77%	0.00%
**Perceived Gender**	Male	52.56%	44.21%
	Female	43.59%	55.79%
	Both	3.85%	0.00%

### Presence versus absence of “do not feed” signs

A GLMM with feeding (yes/no) entered as the dependent variable and presence of sign (present/absent) entered as an explanatory factor revealed that the presence of a sign explained a significant amount of variation in feeding behaviour, with feeding being significantly more likely when a sign was absent (proportion of visitors feeding = 0.020) than present (0.009 X^2^(1) = 10.3, p = .001). In contrast, a second GLMM with trying to touch (yes/no) entered as the dependent variable and sign presence (present/absent) as the explanatory variable revealed visitors were significantly more likely to try and touch the meerkats when a ‘do not feed’ sign was present (proportion of visitors TTT = 0.042) than absent (0.026; X^2^(1) = 3.92, p = .047).

### Sign design: Wording and imagery

A GLMM with feeding (yes/no) entered as the dependent variable and imagery (watching eyes/pawprints) and wording (personal/non-personal) entered as explanatory factors revealed that the design of the sign did not explain a significant amount of variation in feeding behaviour (X^2^(2) = 0.62, p = .732). Equally, a second GLMM revealed the sign wording and imagery did not explain a significant amount of variation in visitors trying to touch the meerkats (X^2^(2) = 0.49, p = .784).

### Factors influencing attention to signs

**Fig 2:**
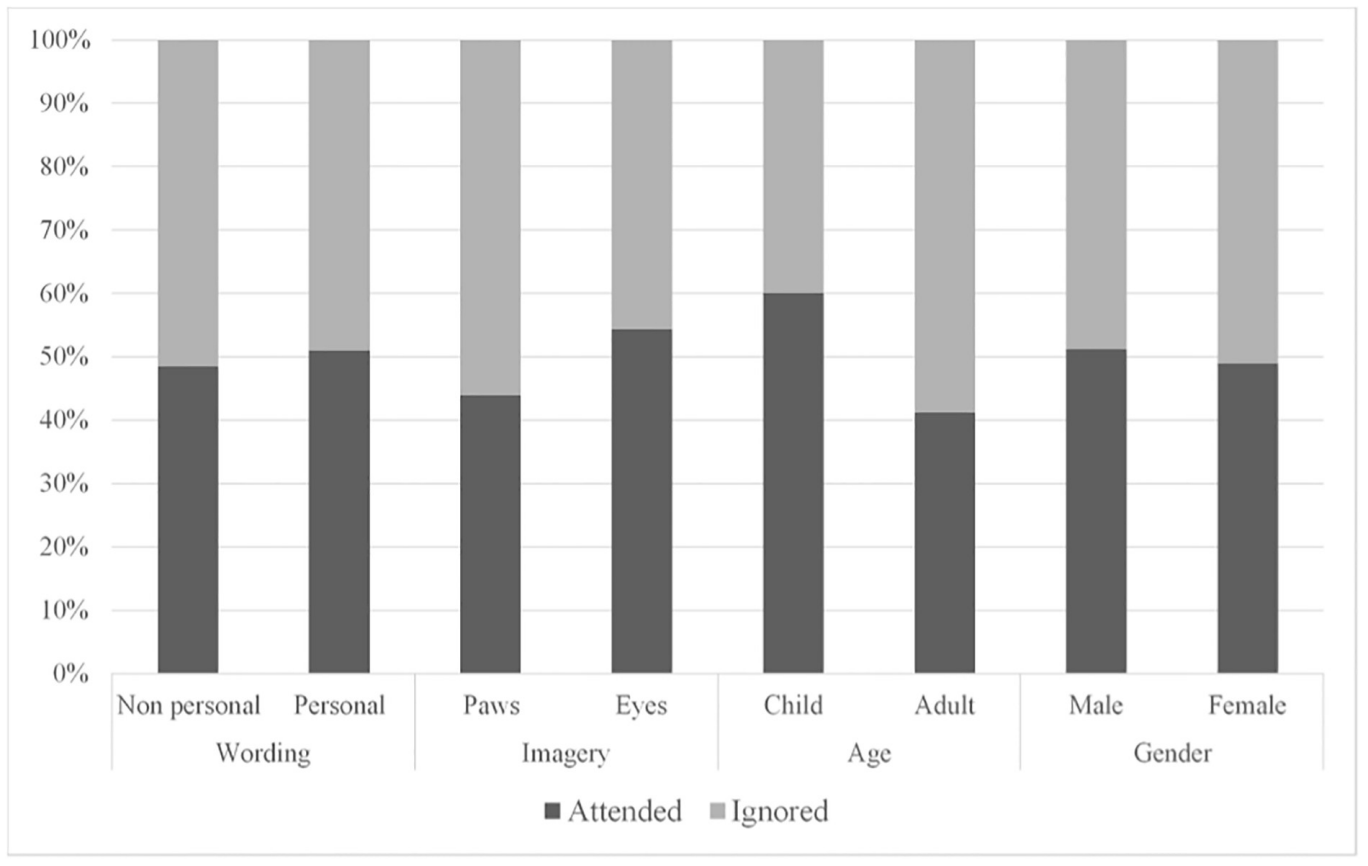
The percentages of visitors attending to the signs, split according to the variables of interest.

A GLMM with attention to sign (attended/did not attend) entered as the dependent variable and imagery (watching eyes/pawprints), wording (personal/non-personal), visitor’s perceived gender (male/female) and visitor age (adult/child) entered as explanatory factors revealed that together these explanatory variables explained a significant amount of variation in visitor attention to the signs (Table 4). When the contribution of individual explanatory variables to the model was considered, age was found to explain a significant amount of variation in visitor attention, with significantly more children (proportion of children attending = 0.068) attending to the sign than adults (0.033; Table 4; Fig 2). In contrast, sign imagery and wording ad and perceived gender of the visitors did not explain a significant amount of variation in visitor attention to the signs (Table 4; Fig 2).

**Table 4:** Results of GLMM testing the influence of sign imagery and wording and visitor age-class and perceived gender on attending to the sign.

Variable	*X*^2^ degrees of freedom	*X*^2^ value	*p*
Overall model	4	17.69	.001
Visitor age-class	1	14.00	< .001
Sign imagery	1	2.24	.133
Sign wording	1	0.57	.452
Perceived gender of visitor	1	0.04	.848

## Discussion

In line with our predictions, the presence of “do not feed” signs containing a pictogram and text were found to reduce feeding but our results suggest trying to touch the meerkats was significantly increased. This could be due to displacement activity from the reduction in the targeted behaviour of feeding. Although the proportion of visitors engaging in feeding was low (0.02), given the large numbers of visitors that visit the enclosure each day (mean = 506), this level of unauthorised feeding poses a considerable health risk to the meerkats in the form an increased chance of obesity or diabetes. Our results indicate that the application of signage can reduce the proportion of visitor who feed the meerkats by approximately half. Although the presence of signs did not entirely eradicate feeding (potentially due to individuals with pre-formed intentions who may possess less awareness of animal welfare and/or rules and regulations), signs were sufficiently effective to warrant future implementation in other enclosures and zoos with similar visitor behaviour problems. Future research would need to assess whether visitor habituation to such signs, if more widely used throughout an institution and for a longer time period, reduces the efficacy of signs and therefore whether strategies need to be implemented to tackle this (e.g. different designs used on different enclosures and renewal of sign design over time).

The failure to find any effect of sign wording on feeding the meerkats indicates that in combination with the pictogram, that both messages are equally effective. It is also possible, however, that due to the efficacy of signs in reducing the proportion of visitors engaging in this behaviour that there was insufficient variation in the data to find an effect hence a larger scale study may be useful. Future research could also investigate whether implementing signs with wording focused on the negative impacts to the visitors themselves (e.g. being bitten), might be more effective than the wording we used.

The failure of watching eyes to further reduce feeding events may also be due to insufficient variation in the data once signs were present. In contrast to previous research that found watching eyes reduced other negative behaviours that were not specified in the sign text [25], visitors did not inhibit attempts to touch the meerkats in the presence of watching eyes images. This could be because visitors don’t perceive this is as a negative behaviour, and therefore were not motivated to inhibit it in order to manage their reputation.

In order to change behaviour, signs need to first capture the visual attention of the audience. Whilst the length of text on the sign and the perceived gender of the visitor did not significantly predict attention to the sign, children were more likely to overtly attend to the sign than adults. Children often read signs aloud to their parents [33] and as our study used reading aloud as a measure of attention, this pattern of results is perhaps not surprising. Our finding is also in line with a previous study that found a higher percentage of children engaged with interpretation signs in a zoo exhibit than adults [34]. Whilst previous studies have argued that watching eyes increase prosocial behaviour through reputation management processes, rather than simply increased visual attention to the sign [25,35], few studies have measured if more participants pay attention to signs with eyes, which is the first necessary step towards adhering with the sign’s message. Our results indicate that visitors were not more likely to pay attention to signs with eyes compared to pawprints.

The implications of the current study are important for both visitors and meerkats as feeding and trying to touch poses health risks to both the meerkats and visitors [3,5]. Our results suggest “do not feed” signs are effective at reducing feeding but attempts to touch the meerkats were increased due to displacement activity. Although only very small proportions of visitors fed or tried to touch the meerkats, the high visitor numbers experienced by Flamingo Land [27] means that seemingly small reductions in the proportion of visitors could represent a high number of visitors whose behaviour would be changed. Based on the mean number of people visiting the enclosure each day during our study, the implementation of “do not feed” signs could prevent over 5 instances of feeding each day.

In conclusion, our study found “do not feed” signs selectively dereased the target behaviour, but increased other negative behaviours. Our findings also indicate that the content of signs should be designed to be accessible to a younger population and have important implications for the wellbeing of both zoo-visitors and zoo-animals. Overall, the current study strongly supports the use of “do not feed” signs in zoos to reduce unauthorised feeding.
